# *Staphylococcus aureus* in Dairy Cattle Milk: Prevalence, Risk Factors, and Antibiogram Profile in Gimbichu Town, Hadiya Zone

**DOI:** 10.1155/vmi/8878629

**Published:** 2026-06-10

**Authors:** Mamo Gobe, Yosef Deneke, Gazali Abafaji, Desta Hail Michael, Eshetu Shumi, S. M. Lutful Kabir, Diriba Taddese

**Affiliations:** ^1^ School of Veterinary Medicine, Jimma University College of Agriculture and Veterinary Medicine, Jimma, Ethiopia; ^2^ Department of Microbiology and Hygiene, Agricultural University, Mymensingh, Bangladesh, cau.edu.cn

**Keywords:** antimicrobials, central Ethiopia, mastitic cows, milk, risk factors

## Abstract

Bovine mastitis, a highly prevalent infectious disease worldwide, frequently involves *Staphylococcus aureus* as a key etiological agent. This cross‐sectional study was conducted from December 2023 to May 2024 in dairy cattle in and around Gimbichu Town, aimed to isolate and identify *S. aureus* from mastitis lactating cows, identify associated risk factors, and determine antibiogram profiles. Milk samples were collected from 384 cows via simple random sampling. Udder/milk examination and the California mastitis test, respectively, diagnosed clinical and subclinical mastitis. The overall mastitis prevalence was 39.06% (6% clinical and 33.06% subclinical). Multivariable analysis identified parity, lactation stage, udder injuries, age, breed, and housing hygiene as significantly associated with mastitis (*p* < 0.05). *S. aureus* was isolated from 42% of mastitis milk, with higher isolation rates in subclinical cases (*p* < 0.01). Antibiogram analysis revealed high resistance to penicillin G, amoxicillin, and tetracycline, with 22.22% of isolates demonstrating resistance to multiple antibiotics across two antimicrobial classes. These findings highlight the significant impact of *S. aureus* mastitis on dairy production in the study area and emphasize the need for targeted prevention and control strategies, including routine antimicrobial susceptibility testing to mitigate the emergence of drug resistance.

## 1. Introduction

Ethiopia’s substantial cattle population (Africa’s largest at 70 million) presents a significant opportunity for milk production. However, the nation faces a persistent challenge in meeting its domestic milk requirements. This is largely attributed to the limited genetic potential of indigenous cattle breeds, which are not as efficient in milk production as specialized dairy breeds. Coupled with this genetic limitation are suboptimal animal husbandry practices, which may include inadequate nutrition, poor housing, and insufficient hygiene [1].

A major contributing factor to reduced milk production is the prevalence of livestock diseases, particularly mastitis. This inflammatory condition of the mammary gland is often caused by bacterial infections, with *Staphylococcus aureus* being a primary culprit [2]. While clinical mastitis is readily apparent, the subclinical form, characterized by the absence of visible symptoms but still resulting in reduced milk yield and altered milk composition, poses a significant yet often overlooked threat to dairy productivity [3, 4].

The persistent underrecognition of mastitis, particularly its subclinical form, underscores the urgent need for strengthened diagnostic capacity and improved management strategies within Ethiopian dairy systems. Effective control of mastitis requires an integrated approach that emphasizes improved milking and housing hygiene, routine screening for subclinical infections, and evidence‐based treatment protocols guided by antimicrobial susceptibility testing. Such measures are critical for reducing production losses, limiting the spread of resistant pathogens, and improving overall milk yield and quality. Addressing mastitis in Ethiopia therefore demands a coordinated, multifaceted strategy that combines genetic improvement of dairy cattle with enhanced husbandry practices and robust disease prevention and control programs [1].

Moreover, antibiotic rising prevalence of *Staphylococcus aureus* (*S. aureus*), particularly methicillin‐resistant (MRSA) and vancomycin‐resistant (VRSA) strains, in livestock has become a significant concern in public health and veterinary medicine [5, 6].

Mastitis drives antibiotic use in Ethiopian dairy herds, increasing antimicrobial resistance (AMR) concerns. While crossbreeding may exacerbate mastitis, a comprehensive *Staphylococcus aureus* assessment in Gimbichu Town, Hadiya Zone, is lacking. Existing research elsewhere does not adequately address local prevalence, risk factors, and AMR profiles.

Characterizing *S. aureus* in Gimbichu Town is crucial. The area is a significant milk producer with many local and crossbred cattle. Local beliefs may delay treatment, and widespread drug use could fuel AMR. While recent Ethiopian studies exist, data on *S. aureus* risk factors and AMR profiles in Gimbichu Town are scarce. This localized investigation aims to provide current susceptibility data for effective veterinary clinical practice.

## 2. Materials and Methods

### 2.1. Description of Study Area

**FIGURE 1:**
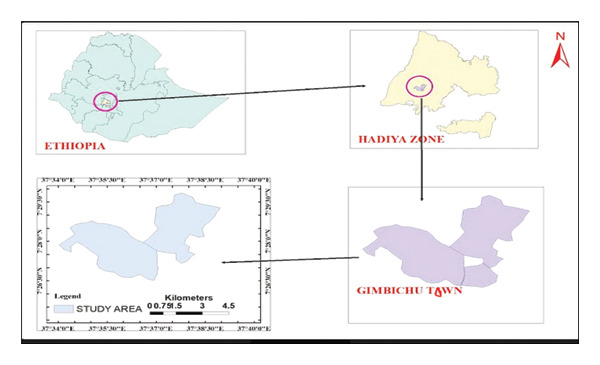
Map of study area (source: https://www.google.com).

This study took place in Gimbichu Town, Hadiya Zone, Central Ethiopia (7°30′‐7°43′ N, 37°35′‐38°05′ E), 296 km south of Addis Ababa. Characterized by mixed farming, Gimbichu receives 1260 mm average rainfall and experiences 19°C average temperature. The town (population ∼35,563) employs intensive/semi‐intensive livestock production using crossbred and local animals, supported by pasture and byproducts. The area sustains a large cattle population (∼25,739) (Figure 1).

### 2.2. Study Design

A cross‐sectional type of study was conducted from December 2023 to May 2024 to isolation and identification of *staphylococcus aureus* from dairy cattle milk, associated risk factors and antibiogram profile test in and around Gimbichu town.

### 2.3. Ethical Approval and Informed Consent

Ethical approval was obtained from the Institutional Animal Care and Use Committee of Jimma University, College of Agriculture and Veterinary Medicine. The study involved noninvasive milk sampling only. Written informed consent was obtained from all dairy farm owners prior to sample collection.

### 2.4. The Study Population

The study population consisted of lactating dairy cows (crossbred and local breeds) in and around Gimbichu town, selected from smallholder dairy farms and households employing intensive and semi‐intensive management systems. Intensive farms housed cattle indoors continuously, providing roughage and concentrates, while semi‐intensive farms allowed outdoor grazing during the day. Age was categorized as young (< 5 years), adult (5–8 years), and old (> 8 years) based on birth records and dentition characteristics. Parity was grouped as few (1‐2 calves), moderate (3–5 calves), and many (> 5 calves). Lactation stage was classified as early (≤ 3 months), mid (> 3–6 months), and late (> 7 months) [7]. Milking hygiene was assessed as good or poor based on premilking udder washing and drying practices. Previous treatment for mammary gland infection was recorded as yes or no [8].

### 2.5. Sample Size and Sampling Technique

The sample size was determined using the formula described by Thrusfield [9], assuming a 95% confidence level and 5% absolute precision. In the absence of previous prevalence data from the study area, an expected prevalence of 50% was used to obtain the maximum required sample size, a conservative and widely accepted approach in epidemiological studies. This assumption is also consistent with reports from comparable dairy production systems in Ethiopia, where prevalence estimates of similar conditions have shown wide variability.

(1) n=1.962pexp1−pexpd2,

where *n* = required sample size; Pexp = expected prevalence.

(2) d2=desired absolute precision.

Accordingly, a total sample size of 384 lactating dairy cows was calculated and proportionally allocated to intensive and semi‐intensive production systems in Gimbichu town and the surrounding Kebeles (Jajjura and Odda). The study areas were purposively selected based on their dairy production potential and livestock population, as recorded by the Gimbichu Town Agricultural Office (7132 registered dairy cows). Farms and households were purposively selected based on the presence of lactating cows and active milk production. Within selected farms, individual lactating cows were chosen using simple random sampling to minimize selection bias at the animal level. Consequently, 116 cows were sampled from Odda, 130 from Jajjura, and 138 from Gimbichu, proportionally reflecting the distribution of the dairy cow population (Table 1).

**TABLE 1:** Proportional allocation of samples.

Study area	Lactating cows	Lactated cows sampled, *n*; calculate size
Odda	2034	116
Jajjura	2381	130
Gimbichu	2717	138
Total	7132	384

### 2.6. Questionnaire Survey

To assess potential risk factors, a semistructured questionnaire was administered, and observational data were collected for 384 lactating cows. Cow‐level factors (breed, age, parity, lactation stage, and pregnancy) and farm‐level factors (herd size, floor type, previous mastitis infection, and towel use) were recorded via owner interviews and direct observation. Milking hygiene (good/poor) was evaluated based on udder hygiene using the methodology of Ruegg [10]. Udder and milk abnormalities (injuries, swelling, clots, and abnormal secretion) were also noted. Finally, drug usage practices in the area were collected from the Gimbichu veterinary clinic to evaluate their potential contribution to AMR patterns.

### 2.7. Clinical Examination and California Mastitis Test (CMT)

Each selected lactating cow was screened for mastitis via clinical examination [4]. Clinical examinations assessed abnormalities in secretion, udder/teats, and systemic reactions. The CMT, performed by mixing 2 mL of milk from each quarter with an equal amount of reagent, was scored based on gel formation within seconds. Scores ranged from negative (0; no gel) to trace (T), weakly positive (+1), distinct positive (+2), and strongly positive (+3). A cow was considered positive for subclinical mastitis if one or more quarters were CMT positive, regardless of microorganism isolation.

### 2.8. Milk Sample Collection

Milk samples were collected aseptically from mastitis‐positive cows that had not been recently or currently treated with antibiotics, in accordance with standard bacteriological procedures [11]. Prior to sampling, teats were thoroughly washed with clean water and dried using disposable paper towels, and the teat ends disinfected with 70% ethanol. The first few streams of milk were discarded to reduce contamination, after which approximately 8–10 mL of milk was collected into sterile, labeled test tubes. Sampling was performed by collecting milk from the near teats first, followed by the far teats, to further minimize cross‐contamination. The samples were transported in an ice box to the Wolaita Sodo Regional Veterinary Laboratory, where they were either immediately inoculated onto appropriate bacteriological media or stored at +4°C overnight prior to culture.

### 2.9. Laboratory Work

#### 2.9.1. Culturing and Biochemical Tests

Milk samples underwent bacteriological examination as per established protocols [11]. Mastitis‐positive samples were cultured using a standardized inoculum volume of 10 μL on 5% sheep blood agar plates (Himedia, India) and incubated aerobically at 37°C for 24 h. Presumptive *Staphylococcus* colonies, based on growth, morphology (golden/yellow color), and β‐hemolysis, were subcultured on nutrient agar (Himedia, India) at 37°C for 24–48 h to obtain pure cultures. Further identification involved Gram staining, morphology assessment, catalase, and oxidase tests. *Staphylococcus aureus* was confirmed through coagulase testing, hemolysis, pigment production (golden‐yellow), and mannitol/maltose fermentation. Quality control was ensured using *Staphylococcus aureus* ATCC 25923. Samples were considered positive for *S. aureus* if at least one colony was identified as such.

#### 2.9.2. Antibiograms Profile Testing

Antimicrobial susceptibility of the *S. aureus* isolates was determined using the Kirby‐Bauer disc diffusion method on Mueller–Hinton agar (Oxoid, England), according to the Clinical and Laboratory Standards Institute [12] and VET08 (2022/2023) guidelines. Antimicrobial agents were selected based on local clinical frequency and availability. The panel of antibiotic discs (Himedia, India) included chloramphenicol (10 μg), gentamicin (30 units), cefoxitin (30 μg), cefotaxime (10 μg), amoxicillin (20 μg), sulfamethoxazole/trimethoprim (300 units), penicillin‐G (10 units), and tetracycline (30 μg).

Bacterial suspensions were prepared from freshly grown nutrient agar cultures using sterile normal saline. The turbidity of each suspension was standardized to 0.5 McFarland using a densitometer, and the inoculum was evenly swabbed onto Mueller–Hinton agar plates with sterile cotton swabs. Following disc placement, plates were incubated at 37°C for 24 h. Inhibition zone diameters were measured and interpreted as susceptible, intermediate, or resistant. Cefoxitin was utilized as a surrogate marker for methicillin resistance. Multidrug resistance (MDR) was defined as resistance to at least one agent in three or more antimicrobial classes according to Magiorakos et al. [13]. Chloramphenicol was included solely for epidemiological comparison due to its restricted use in food‐producing animals.

### 2.10. Data Management and Statistical Analysis

Data were managed and descriptive statistics computed using Microsoft Excel. Following data entry and validation in Excel, SPSS Version 20.0 was used for analysis. Pearson’s chi‐square (*χ* ^2^) tests were used to assess associations between categorical variables and *S. aureus* mastitis risk factors. A chi‐square value was used to further examine the association of potential risk factors with mastitis. Significance was set at *p* < 0.05 for all analyses.

## 3. Results

### 3.1. Prevalence of *S. aureus*

A total of 384 lactating cows were examined, of which 150 (39.06%) were positive for mastitis. Among the mastitis cases, 23 (6%) were clinical, and 127 (33.07%) were subclinical. Milk samples collected from all 150 mastitis positive cows were subjected to microbiological analysis. *Staphylococcus aureus* was isolated from 9 (39.1%) of the clinical cases and 54 (42.5%) of the subclinical cases, yielding an overall *S. aureus* prevalence of 42% (63/150) among mastitis positive cows. A statistically significant difference (*p* < 0.05) was observed in the distribution of *S. aureus* between clinical and subclinical mastitis cases (Table 2).

**TABLE 2:** Prevalence of *Staphylococcus aureus* in subclinical and clinical mastitis.

Forms of mastitis	No. of animal’s positive	No. of *S. aureus* isolated	Prevalence of *S. aureus*	*χ* ^2^	*p* values
Subclinical	127	54	33.07%		
Clinical	23	9	6%	0300.57	≤ 0.01
Total	150	63	42%		

Across the three study areas, 384 lactating cows were examined, comprising 116 from Odda, 130 from Jajjura, and 138 from Gimbichu. The prevalence of mastitis was 29.3% (34/116) in Odda, 36.9% (48/130) in Jajjura, and 52.3% (72/138) in Gimbichu (Table 3).

**TABLE 3:** Distribution and Prevalence of Clinical and Subclinical Mastitis Across Study Areas.

Study area	Examined	Positives study area		Prevalence (%)
		Subclinical (%)	Clinical (%)	
Odda	116	30 (25.86)	4 (3.44)	34 (29.31)
Jajjura	130	41 (31.53)	7 (5.38)	48 (36.92)
Gimbichu	138	56 (40.57)	12 (8.69)	68 (52.30)
Total	384	127 (33.07)	23 (6)	150 (39.06)

### 3.2. Risk Factors Associated With Mastitis Prevalence

The questionnaire survey and observation data result shows in the present study parity, stage of lactation, udder injuries, age, breeds and house hygiene had significant association with the prevalence of clinical and subclinical mastitis (P < 0.05). Lactating cows with above 8 years age are highly infected, while those at the age interval of 5–8 years are less susceptible (*p* < 0.05); likewise, prevalence was significantly high in cows with parity number more than 5 and less in those with parity 4 up to 5 (*p* < 0.05). Unlike early stage lactation, cows in late‐stage lactation suffered (*p* < 0.05). Infection is high in cows with injured udder/teat (*p* < 0.05). Cows which are managed in poor house condition showed significantly high prevalence (*p* < 0.05). However, milkers hand hygiene, udder hygiene, and common towel used for drying teat did not show statistically significant (*p* > 0.05) variations (Table 4).

**TABLE 4:** Risk factors associated with Mastitis.

Risk factor	Total no. of examine	No. of positive prevalence (%)	*χ* ^2^	*p* values
Breed				
Cross	104	48 (46.15%)	4.014 a	0.045
Local	46	15 (32.6%)		
Age				
3–5 years	50	21 (42%)	18.773 a	0.001
6–8 years	76	18 (23.68%)		
> 8 years	24	24 (100%)		
Lactation stage				
1–3 months	58	15 (25.86%)	15.171 a	0.001
4–5 months	65	22 (33.84%)		
> 5 months	27	26 (96.3%)		
Parity				
1–3 birth	85	35 (62.5%)	10.098 a	0.001
4‐5 birth	56	19 (22.35%)		
Over 5 births	9	9 (100%)		
Udder/teat condition				
Not injured	123	37 (30.08%)	13.468 a	0.001
Injured	27	26 (96.3%)		
Udder hygiene				
Poor hygiene	75	36 (48%)	2.046 a	0.121
Good hygiene	75	27 (36%)		
Hand hygiene before milking				
Washed	72	25 (34.72%)	3.627 a	0.057
Not washed	78	38 (48.71%)		
Usage of towel				
Common towel	39	25 (64.10%)	0.789 a	0.375
No use of towel	111	38 (34.2%)		
House hygiene				
Good	70	17 (24.28%)	20.798a	0.001
Poor	80	46 (57.5%)		

### 3.3. Antibiograms Profile Testing

All the isolated sixty‐three *S. aureus* were tested for susceptibility to selected antibiotics. Antibiograms used in this study were amoxicillin, sulphamethoxazole/trimethoprim, tetracycline, chloramphenicol, cefoxitin, cefotaxime, gentamicin, and penicillin G.

Present study has demonstrated the existence of the levels of resistance of *S. aureus* in the study area in 100 (63/63) to penicillin, 100 (63/63) to amoxicillin, 80.95 (51/63) to tetracycline, and 4.76 (3/63) to cefoxitin. *S. aureus* isolates were susceptible to sulphamethoxazole/trimethoprim, gentamicin, and chloramphenicol (Table 5).

**TABLE 5:** Antibiograms profile test on isolated *S. aureus*.

Antibiotics tested	Susceptible (%)	Intermediate (%)	Resistant (%)
Penicillin G	—	—	100 (63/63)
Cefotaxime(CXT)	77.78 (49/63)	22.22 (14/63)	—
Cefoxitin (FOX)	95.23 (60/63)	—	4.76 (3/63)
Sulphamethoxazole/trimethoprim	100 (63/63)	—	—
Tetracycline (TE)	14.28 (9/63)	4.76 (3/63)‐	80.95 (51/63)
Gentamicin (CN)	100 (63/63)	—	—
Chloramphenicol (C)	100 (63/63)	—	—
Amoxicillin	—	—	100 (63/63)

Of the resistant *S. aureus* isolates, 22.22% (14/63) exhibited resistance to four antimicrobial agents’ penicillin G, amoxicillin, cefoxitin, and tetracycline. However, as these antibiotics represent resistance to only two antimicrobial classes (β‐lactams and tetracyclines), the isolates do not meet the standard criteria for MDR.

## 4. Discussion

The present study revealed an overall *Staphylococcus aureus* prevalence of 42% among mastitis cows in the study area. This finding is comparable with reports from Adama (42.1% [8]), Southern Ethiopia (40.3% [14]), Asella (39.1%), Shinshicho (38.75% [15]), and Haramaya University and its surroundings (66.0% [2]). However, it exceeds earlier reports from Southern Ethiopia (23% [16]) and Bahir Dar (28.2% [17]), while remaining lower than reports from Asella (44.62% [18]) and Assosa (46.5% [19]). Such variations likely reflect differences in herd management, milking hygiene, environmental conditions, antimicrobial usage practices, and levels of awareness regarding mastitis transmission. As a contagious pathogen, *S. aureus* is primarily transmitted during milking through contaminated hands, towels, utensils, and close animal contact, particularly under poor hygienic conditions [4].

In the present study, the prevalence of subclinical mastitis (33.07%) was markedly higher than that of clinical mastitis (6%), a finding consistent with previous reports from Adama (36.7% subclinical and 10% clinical [8]). This consistent predominance of subclinical mastitis across studies may be attributed to the lack of obvious clinical signs, leading to underrecognition by farmers and delayed or absent treatment. In Ethiopia, control efforts have traditionally focused on clinical mastitis, while subclinical cases often remain untreated, resulting in persistent intramammary infections and sustained production losses [20].

Breed was significantly associated with mastitis prevalence, with crossbred cows showing higher infection rates than local breeds, in agreement with findings by Mulugeta and Wassie [21]. This may be explained by the larger, pendulous udders of crossbred cows, which are more prone to environmental contamination and injury. Moreover, intensive genetic selection for higher milk yield may be accompanied by increased susceptibility to mastitis. In contrast, the lower prevalence observed in local breeds may be related to stronger genetically determined physical barriers, such as teat canal structure and sphincter function, as well as differences in innate and cellular immunity [22].

Age was another significant risk factor, with higher prevalence observed in older cows, consistent with reports by Haftu et al. [23] and Kerro and Tareke [14]. This trend likely reflects cumulative exposure to pathogens over time, repeated milking stress, and progressive weakening of teat canal defenses, which facilitate pathogen entry [3, 4]. Similarly, mastitis prevalence increased with advancing lactation stage, particularly during late lactation, in line with findings by Getahun et al. [24] and Haftu et al. [23]. Although some studies have reported higher susceptibility during early lactation [14], such discrepancies may be due to differences in breed composition, parity, management practices, and herd health programs across study settings.

Parity was also significantly associated with mastitis occurrence, with higher prevalence observed in multiparous cows, particularly those with more than five calvings. This agrees with reports by Erskine [22], who attributed increased susceptibility in multiparous cows to reduced teat sphincter tone, increased teat canal patency, and repeated pathogen exposure. Variations observed between parity groups in this study may additionally reflect differences in breed, housing, and milking management [4].

Teat and udder injuries were strongly associated with mastitis, with a very high prevalence observed among injured cows. Such injuries, often caused by barbed wire fences, tick infestation, or aggressive suckling, provide favorable entry points for bacterial invasion and secondary infections. Infected udders serve as the primary reservoir of *S. aureus*, with transmission occurring via milkers’ hands, equipment, towels, and the housing environment [4]. Poor hand hygiene among milkers has been repeatedly identified as a major contributor to *S. aureus* transmission [25].

House hygiene significantly influenced mastitis prevalence, with markedly higher rates observed in poorly managed housing systems. Accumulation of manure and wet bedding creates an environment conducive to bacterial survival and transmission, a finding consistent with earlier Ethiopian studies that linked poor barn hygiene with increased mastitis prevalence [21, 26].

The present study detected *S. aureus* in 39.1% of clinical and 42.5% of subclinical mastitis cases. The relatively higher proportion in subclinical mastitis may reflect the organism’s ability to establish chronic, persistent intramammary infections and the frequent lack of treatment for subclinical cases. In the broader Ethiopian context, chronic *S. aureus* infections have been associated with increased severity of udder damage and reduced treatment success, particularly when infections involve multidrug‐resistant (MDR) strains.

Although the present study did not evaluate mastitis severity scores in relation to MDR status or treatment outcomes, previous reports from central and southeastern Ethiopia have documented reduced therapeutic efficacy and prolonged infection duration in MDR *Staphylococcus aureus* cases compared with nonresistant strains [18, 27]. These findings underscore the clinical significance of AMR, extending beyond prevalence estimates to its impact on disease persistence and treatment success.

Antimicrobial susceptibility testing showed that *S. aureus* isolates were most susceptible to chloramphenicol, gentamicin, and sulfamethoxazole/trimethoprim, consistent with findings from other Ethiopian studies [8, 18, 28]. However, high levels of resistance were observed against penicillin G (100%), amoxicillin (100%), and tetracycline (80.95%), in agreement with reports from Kombolcha [7], Ambo and Gudar, Arsi [27], and similar studies from other countries [29]. This widespread resistance is likely driven by prolonged and indiscriminate use of these commonly available antimicrobials.

The complete resistance to penicillin G is particularly concerning, as this drug is frequently recommended for the treatment of staphylococcal mastitis. Resistance is commonly mediated by beta‐lactamase production, which inactivates penicillin and related β‐lactam antibiotics [30]. Penicillin resistance is often considered a marker for reduced susceptibility to other β‐lactams [31, 32]. Frequent use of penicillin and amoxicillin for intramammary infections in the study area may have contributed to the observed resistance, as also reported by Kemal et al. [18]. Approximately half of mastitis‐causing *S. aureus* strains are known to produce beta‐lactamase, resulting in poor treatment outcomes and persistent infections [33, 34]. These findings underscore the importance of routine antimicrobial susceptibility testing prior to treatment to minimize therapeutic failure and reduce the public health risk associated with resistant strains [35].

The occurrence of AMR varied among isolates, reflecting differences in antibiotic exposure at herd and farm levels, as previously noted by Pace [32]. Based on the present susceptibility profile, gentamicin and sulfamethoxazole/trimethoprim appear to be the most effective options for treating *S. aureus* mastitis in the study area; however, their use should be guided by susceptibility testing to prevent further resistance development.

Finally, several limitations of this study should be acknowledged. Although the cross‐sectional design is appropriate for estimating prevalence and identifying associations, it does not allow for causal inference between risk factors and mastitis occurrence. In addition, the purposive selection of farms prior to random selection of individual cows may have introduced selection bias, potentially affecting the generalizability of the findings to the wider dairy population. Furthermore, the study did not assess mastitis severity in relation to MDR status, nor did it evaluate treatment outcomes. Future longitudinal studies incorporating clinical severity scoring, molecular characterization of resistance, and follow‐up of treatment responses are recommended to better elucidate the epidemiological and clinical impact of MDR *S. aureus* mastitis in Ethiopia.

## 5. Conclusion and Recommendations

Mastitis significantly constrains dairy production in intensive and semi‐intensive systems, with management factors playing a crucial role in its control. This study identified parity, lactation stage, udder injuries, age, breed, and animal hygiene as prominent risk factors in the study area, with crossbred cows being more affected. The high prevalence of *S. aureus* mastitis underscores its impact on milk production. A concerning level of resistance to penicillin G, tetracycline, and amoxicillin was observed, likely linked to their predominant use in treating mastitis and other infections, as evidenced by veterinary clinic data. The susceptibility to trimethoprim, gentamicin, and chloramphenicol suggests their infrequent use for mastitis treatment. Therefore, we recommend strict adherence to milking hygiene protocols, prompt treatment of infected animals, proper hand hygiene for milkers, careful consideration of animal history (age, lactation stage, and parity) before introduction to herds, and regular screening with bacteriological examination to guide appropriate drug administration. Implementation of systematic mastitis control methods, including antibiotic susceptibility testing prior to any antibiotic use, is crucial to combatting *S. aureus* mastitis and preserving antibiotic efficacy.

## Funding

No funding was received for this manuscript.

## Conflicts of Interest

The authors declare no conflicts of interest.

## Data Availability

The data that support the findings of this study are available on request from the corresponding author. The data are not publicly available due to privacy or ethical restrictions.
